# Optimization of Somatic Embryogenesis and Transcriptomic Analysis of the Early Stage of Callus Redifferentiation in *Quercus suber* L.

**DOI:** 10.3390/plants14182855

**Published:** 2025-09-12

**Authors:** Xinran Yu, Yaru Hou, Wan Zhang, Han Gong, Baoxuan Liu, Xiaozhou Song, Tiezhu Li, Yun Yang, Jingle Zhu

**Affiliations:** 1 Research Institute of Non-Timber Forestry, Chinese Academy of Forestry, 3 Weiwu Road, Zhengzhou 450003, China; 2 College of Forestry, Henan Agricultural University, 63 Agricultural Road, Zhengzhou 450002, China; 3 Hainan Key Laboratory of Tropical Oil Crops Biology, Coconut Research Institute, Chinese Academy of Tropical Agricultural Sciences, Wenchang 571339, China; 4 Shandong Cork Oak Industrial Technology Research Institute Co., Ltd., Jiazhuang Village, Liying Street, Rencheng District, Jining 272075, China; 5 College of Forestry, Northwest A&F University, 3 Taicheng Road, Xianyang 712100, China; 6 College of Biological and Food Engineering, Huanghuai University, Kaiyuan Road, Zhumadian 463000, China

**Keywords:** *Quercus suber*, somatic embryogenesis, tissue culture, transcriptomics

## Abstract

*Quercus suber* L. (*Q. suber*) is an ecologically and industrially valuable species, yet faces challenges in propagation in China. This study optimized somatic embryogenesis (SE) protocols using two-year-old *Q. suber* leaves, focusing on petioles and leaf veins as the most responsive explants, with May as the optimal sampling time. The MSSH medium (a combination of Murashige and Skoog Medium (MS) major elements and Schenk and Hildebrandt Medium (SH) minor elements and vitamins) under darkness maximized transdifferentiation. Additionally, the highest callus induction was achieved with 0.50 mg/L 6-benzyladenine (6-BA) and 1.00 mg/L 1-Naphthaleneacetic acid (NAA). Liquid culture with 1.00 g inoculum and 0.50 mg/L 6-BA + 0.20 mg/L NAA achieved the best proliferation. Redifferentiation peaked at 0.15 mg/L NAA + 0.20 mg/L 6-BA. Transcriptome profiling identified 4534 differentially expressed genes (DEGs) between embryogenic callus (E1) and global embryos (E2), with key pathways linked to cell wall remodeling, stress responses, and photosynthesis. Key regulators identified during the early stage of callus redifferentiation include cytokinin oxidase 3 (*CKX3*), gibberellin-responsive protein (*GH3.6*), and pectin lyase 5 (*PL5*), among others. This study provides insights into efficient SE of *Q. suber* and the genes underlying early callus redifferentiation, laying the groundwork for future research.

## 1. Introduction

*Quercus suber* L. (*Q. suber*), commonly known as the “cork oak”, is a tree species in the Fagaceae family and *Quercus* genus, recognized for its well-developed cork tissue [1]. The cork of *Q. suber* has excellent elasticity, waterproof properties, thermal insulation, and micro-permeability, playing an important role in various fields such as cork stopper production, aerospace materials, and more [2,3,4]. As a result, the demand for *Q. suber* has been increasing. The species has been widely introduced around the world, with multiple batches of introduction efforts carried out in China [5]. Unfortunately, despite some successful plantings, there have been no large-scale success cases. Currently, only one *Q. suber* is preserved in China, with a forest age of 60 years [6,7,8]. Given that this genotype is adapted to the climate of Hubei Province in China, its conservation and propagation are of great significance. However, the low rooting success rate of cuttings, weak growth, limited seed production, and difficulty in long-term seed storage have restricted its conservation and utilization [9].

Vegetative reproduction offers advantages such as rapid reproduction, high genetic stability, and minimal environmental influence [10], making it an important method for the propagation of many economic crops. However, the low clonal propagation ability of *Quercus* species [11] has limited the widespread application of this technique. Somatic embryogenesis (SE) can be classified into direct and indirect development. Direct SE refers to the development of somatic embryos or embryogenic tissues directly from the explant, bypassing the intermediate callus stage [12]. In indirect SE, plant organs or tissues are excised as explants and induced to undergo transdifferentiation, leading to the formation of an intermediate cell mass known as callus. This process has often been described in the literature as either dedifferentiation or transdifferentiation [13,14], although the definitions of these terms remain debated. Dedifferentiation is generally considered to occur only within the same developmental lineage, whereas transdifferentiation denotes cell fate changes that occur independently of developmental potency. Because callus formation is not a simple regression along a developmental lineage but rather results from excessive proliferation and fate transition, many scholars argue that it should be considered a form of transdifferentiation [15]. Upon further adjustment of induction conditions, a new stem cell region (meristem) is formed, which is referred to as embryogenic callus [16]. The embryogenic callus subsequently undergoes redifferentiation—the process by which undifferentiated or dedifferentiated cells regain specialized functions [17]—to form embryoids, including global, heart-shape, torpedo-shape, cytoledon-stage embryo. This is followed by embryo maturation and the induction of embryo germination, ultimately giving rise to complete plants. This technique has significantly improved the efficiency of in vitro regeneration in various plant species [18]. In this study, we use the term transdifferentiation to describe the induction process by which explant-derived cells undergo a fate change and form callus tissue. The subsequent transition from embryogenic callus to organized embryoids is referred to as redifferentiation. This terminology is adopted to provide conceptual clarity and to avoid confusion in describing the sequential stages of somatic embryogenesis. SE is characterized by relatively stable genetic traits in offspring and has been widely applied in seedling production, molecular breeding, and plant improvement [19]. During SE in plant tissue culture, callus is first formed through transdifferentiation, followed by the induction of embryogenic callus, and finally redifferentiation results in the formation of somatic embryos. These three stages are regulated by multiple factors, among which the type of explant, the composition of the culture medium, and the supplementation with plant growth regulators (PGRs) represent the most critical determinants [20,21,22]. Studies have demonstrated that ovaries, ovules, and immature zygotic embryos are the most effective explants for SE [23]. The developmental age of the explant significantly influences the transcriptional regulatory network governing somatic embryo development [24]. The formation of globular embryos (E2) from embryogenic callus (E1) marks an early stage of redifferentiation [25], serving as a critical transition point in somatic embryo formation. Previous studies have established SE technology for *Q. suber* [26], primarily focusing on immature zygotic embryos [27,28], mature zygotic embryos [29], and mature leaf explants [30] from *Q. suber* in Southern Europe. However, research on the induction of SE from Chinese-origin *Q. suber* seedlings remains scarce. In terms of culture medium, several basal formulations have been widely used for SE, including Murashige and Skoog (MS), Schenk and Hildebrandt (SH), Gamborg’s B5, and Driver and Kuniyuki walnut (DKW) media [31,32,33]. In addition, modified media have been shown to improve callus induction and embryogenic competence in recalcitrant species [34]. PGRs also play a decisive role in regulating SE. The balance between auxins (such as 2,4-dichlorophenoxyacetic acid (2,4-D) and 1-Naphthaleneacetic acid (NAA)) and cytokinins (such as 6-benzyladenine (6-BA) and kinetin (KT)) is particularly critical, where low cytokinin-to-auxin ratios typically favor callus induction, whereas higher ratios promote somatic embryo differentiation and maturation [35,36]. Moreover, gibberellins, abscisic acid, and polyamines have been reported to further modulate embryo maturation and conversion efficiency in woody plants [37,38]. In previous studies, somatic embryogenesis of *Q. suber* has been successfully induced on MS, 1/2 MS, and SH basal media, with frequent supplementation of plant growth regulators such as 6-BA, 2,4-D, and zeatin (ZT) in various combinations [30,39,40]. These combinations have been reported to promote both callus induction and embryogenic competence, although the optimal formulation often varies depending on the developmental stage of the explant and the genotype of the donor plant.

SE is influenced not only by culture conditions but also by the intrinsic regulation of signal transduction and gene expression [41,42]. Transcriptome analysis, a widely used high-throughput sequencing technique, plays a crucial role in identifying key genes and exploring molecular mechanisms. Numerous studies have investigated the molecular mechanisms of SE across various plant species, revealing that it is regulated by differentially expressed genes (DEGs) and transcription factors (TFs), such as *LEAFY COTYLEDON* (*LEC*), *BABY BOOM* (*BBM*), and *SOMATIC EMBRYOGENESIS RECEPTOR KINASE* gene (*SERK*) [43]. In *Q. suber*, transcriptomic research has primarily focused on cork formation [44], unisexual flower development [45], and key developmental stages of SE [46] in Southern Europe. However, studies on the gene expression processes during callus formation remain limited, particularly in relation to identifying somatic embryo markers and analyzing the molecular mechanisms behind redifferentiation. By analyzing the gene expression patterns during callus redifferentiation via transcriptome analysis, it is expected that key signaling pathways and regulatory factors involved in SE can be identified, providing theoretical support for predicting molecular markers for somatic embryo formation in the future. Additionally, studies have shown that callus transcriptome data from various species share certain similarities. For instance, genes associated with callus redifferentiation have been identified in species such as tomato [47] and kiwifruit [48], providing a strong foundation for comparative analyses across species.

Based on the method developed by Vladimír Chalupa [49] for inducing SE in *Q. suber* leaves and successfully cultivating plants, this study uses 2-year-old seedling leaves of *Q. suber* grown in China for SE induction and further optimizes the technique. By analyzing gene expression pattern differences during the early stages of callus redifferentiation in *Q. suber*, the study explores the regulatory pathways involved in this process and identifies key genes and transcription factors. The results of this study lay a foundation for improving the clonal propagation rate of *Q. suber* and provide new insights for research on the molecular mechanisms of SE and functional gene validation in *Q. suber*.

## 2. Results

### 2.1. Transdifferentiation to Form Callus

#### 2.1.1. Effects of Different Explants on Transdifferentiation to Form Callus of *Q. suber*

**Figure 1:**
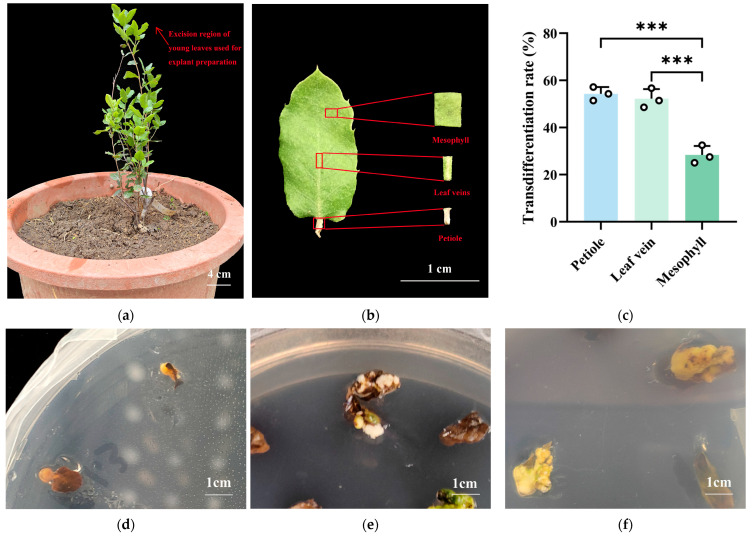
(**a**) Two-year-old *Q. suber* plant showing the excision regions of young leaves (indicated by arrows); (**b**) Photograph of a *Q. suber* leaf and schematic representation of the excised explants, including mesophyll, leaf veins, and petiole.; (**c**) Transdifferentiation rates of different explants (*N* = 45, *R* = 3, All data are shown as mean ± SD. *** *p* < 0.001); Callus formation induced by leaf veins (**d**), mesophyll (**e**), and petioles (**f**).

Immature leaves were harvested from a two-year-old *Q. suber* plant (Figure 1a). Notably, transdifferentiation efficiency varied significantly among the different types of explants (Figure 1b,c). The transdifferentiation rate was significantly higher in petioles and leaf veins compared to the mesophyll, reaching up to 54.29%. The callus derived from petioles and leaf veins was mostly yellowish-white and spherical in shape (Figure 1d,f). After sterilization, the mesophyll turned brown, and the callus was mostly white, with irregular shape, making it prone to death (Figure 1e). Therefore, petioles and leaf veins of *Q. suber* leaves are the most suitable explants for leaf transdifferentiation to form callus, and the callus was used for subsequent somatic embryo induction.

#### 2.1.2. Effects of Sampling Time on Transdifferentiation to Form Callus of *Q. suber*

The transdifferentiation rate of the explants varied with the time of sampling, and the growth status of the resulting callus also differed (Table 1). The highest transdifferentiation rate, 62.22%, was observed in explants collected in May, with the callus being yellowish-white and growing vigorously. Explants collected after August showed gradual browning. The callus from explants collected in November and January of the following year was white, with a significant decrease in the transdifferentiation rate, reaching a low of 7.41%. Therefore, the optimal time for explant collection is May.

#### 2.1.3. Effects of Basic Mediums, Light Conditions and PGRs on Transdifferentiation to Form Callus of *Q. suber*

**Figure 2:**
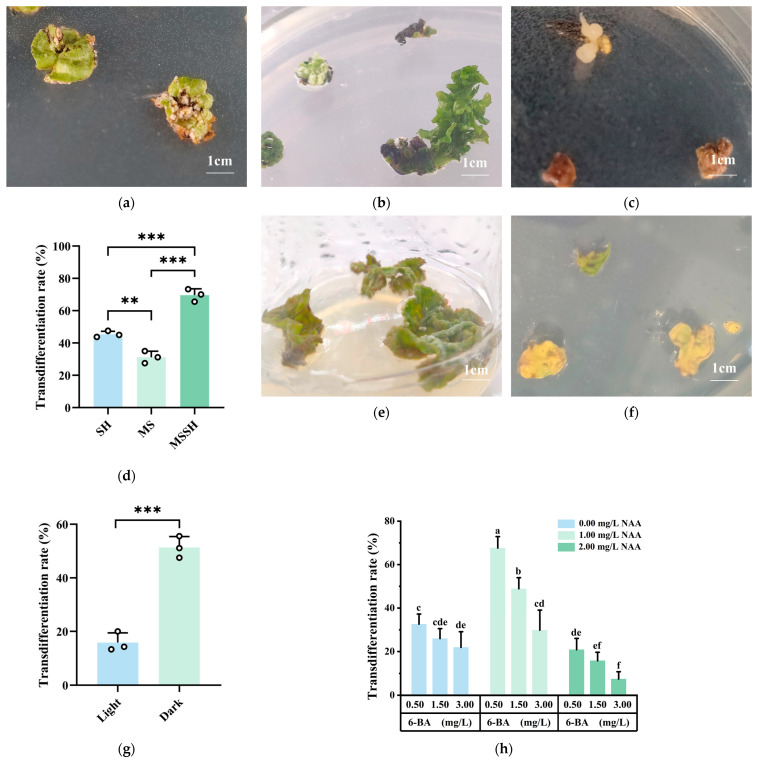
Effects of basic mediums, light conditions, and PGRs on transdifferentiation to form callus of *Q. suber*: Growth of explants on SH basic medium (**a**), MS basic medium (**b**), MSSH basic medium (**c**); (**d**) Transdifferentiation rate of different basic mediums; Growth of explants under light (**e**), dark (**f**) conditions; (**g**) Transdifferentiation rate of different light conditions when cultured on MSSH medium; (**h**) Transdifferentiation rate of different PGRs. (Means ± SD error within a column. Different letters meant there was significant difference among groups, *p*  <  0.05 (*N* = 45, *R* = 3, ** *p* < 0.01, *** *p* < 0.001).

Explants were able to form callus on different basic mediums, but the transdifferentiation rates differed significantly, with varying growth conditions. The explants grew slowly on the SH basic medium, and rust-colored callus formed at the leaf edges (Figure 2a). The transdifferentiation rate was low at 45.52%. On the MS basic medium, dense white callus formed at the leaf veins, with a few explants showing direct organogenesis (Figure 2b). The transdifferentiation rate was the lowest at 31.25%. On the MS major elements + SH minor elements and vitamins (MSSH) basic medium, the explants grew faster, with yellowish-white transparent callus forming at the leaf veins and petioles, and a few explants exhibiting direct SE (Figure 2c). The transdifferentiation rate was the highest at 69.93% (Figure 2d). Therefore, MSSH was the most suitable basic medium for inducing transdifferentiation to form callus of explants.

Explants were cultured under both light and dark conditions, with dark incubation significantly promoting transdifferentiation to form callus. Under light conditions, the explants primarily formed green callus (Figure 2e), with a transdifferentiation rate of 15.87%. In the dark, the explants predominantly formed yellowish-white callus (Figure 2f), with the highest transdifferentiation rate of 51.39% (Figure 2g). Therefore, the most suitable light condition for inducing transdifferentiation to form callus was darkness.

Explants showed significant differences in transdifferentiation rates when cultured on media containing different PGRs combinations. With a constant NAA concentration, the transdifferentiation rate decreased as the 6-BA concentration increased. The transdifferentiation rate of explants treated with 0.50 mg/L 6-BA was significantly higher than that of explants treated with different concentrations of 6-BA at the same NAA concentration. With a constant 6-BA concentration, the transdifferentiation rate of explants increased and then decreased as the NAA concentration increased. The transdifferentiation rate of explants treated with 1.00 mg/L NAA was significantly higher compared to explants treated with other NAA concentrations at the same 6-BA concentration. The highest transdifferentiation rate (67.62%) was obtained with 0.50 mg/L 6-BA + 1.00 mg/L NAA (Figure 2h). Therefore, the most suitable plant growth regulator combination for inducing explant transdifferentiation was 0.50 mg/L 6-BA + 1.00 mg/L NAA.

### 2.2. Callus Proliferation

**Figure 3:**
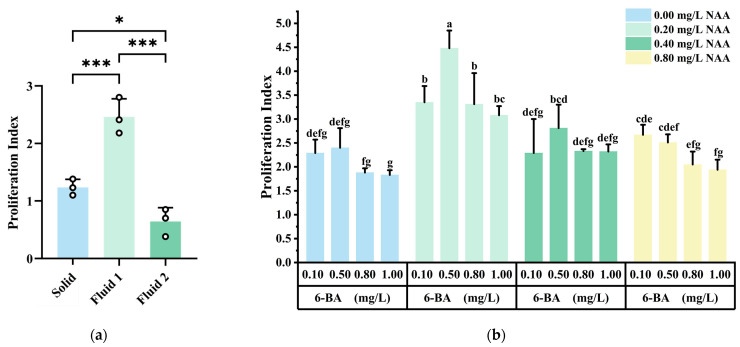
Effects of culture methods and PGRs on subculture of *Q. suber*: (**a**) Proliferation index of embryonic callus under different culture conditions: Solid medium (Solid), liquid medium with 1.00 g inoculum (Fluid 1), and liquid medium with 3.00 g inoculum (Fluid 2).; (**b**) Proliferation index of embryonic callus under different PGRs (Means ± SD error within a column. Different letters meant there was significant difference among groups, *p*  <  0.05. *N* = 45, *R* = 3, * *p* < 0.05; *** *p* < 0.001).

Liquid culture is commonly used for callus subculture and proliferation. In this experiment, embryogenic callus was cultured both in liquid and solid mediums. As shown in Figure 3a, the proliferation rate of callus in liquid medium with 1.00 g inoculum (Fluid 1) was significantly higher than in solid medium (Solid) (*p* ≤ 0.001). However, when 3.00 g of callus was used in liquid medium (Fluid 2), the proliferation rate decreased significantly (*p* ≤ 0.001), and the callus showed slight browning. Therefore, liquid culture with 1.00 g inoculum was identified as the most suitable condition for callus proliferation.

The proliferation of embryogenic callus varied under different PGRs combinations (Figure 3b). When the NAA concentration was constant, the proliferation rate first increased and then decreased as the 6-BA concentration increased, with the best proliferation observed at 0.50 mg/L 6-BA. High concentrations of 6-BA (0.80 mg/L, 1.00 mg/L) inhibited the proliferation of embryogenic callus. When the 6-BA concentration was constant, the proliferation rate first increased and then decreased as the NAA concentration increased. The proliferation rate of embryogenic callus treated with 0.20 mg/L NAA was significantly higher than that of other treatments. The highest proliferation rate of 4.48 times was observed when treated with 0.50 mg/L 6-BA + 0.20 mg/L NAA. Therefore, the most suitable PGRs combination for callus subculture and proliferation was 0.50 mg/L 6-BA + 0.20 mg/L NAA.

### 2.3. Induction, Maturation, and Germination in Somatic Embryogenesis

**Figure 4:**
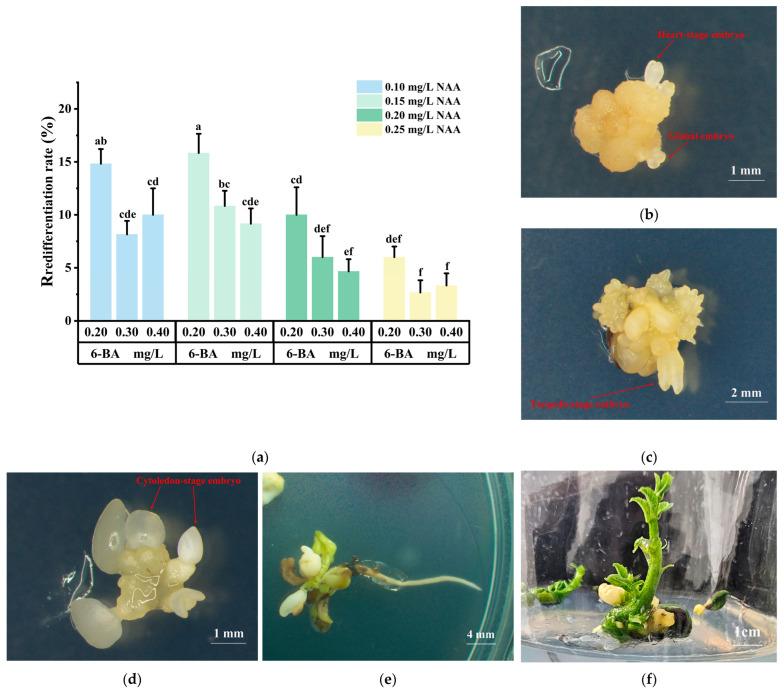
Effects of PGRs on redifferentiation of *Q. suber*: (**a**) Redifferentiation rate of embryonic callus under different plant growth regulators; (**b**) Global embryo and heart-stage embryo; (**c**) Torpedo-stage embryo; (**d**) Cytoledon-stage embryo; (**e**) The mature cotyledon-stage embryo after being transferred to light; (**f**) Plantlets regenerated from somatic embryos (Means ± SD error within a column. Different letters meant there was significant difference among groups, *p* < 0.05. *N* = 45, *R* = 3).

Embryogenic callus was able to redifferentiate into somatic embryos on media supplemented with different plant growth regulator combinations. However, significant differences in redifferentiation rates were observed (Figure 4a). When the NAA concentration was fixed, the redifferentiation rate decreased as the 6-BA concentration increased, with low concentrations of 6-BA (0.20 mg/L) inducing redifferentiation in the embryogenic callus. When the 6-BA concentration was fixed, the redifferentiation rate first increased and then decreased as the NAA concentration increased. The highest redifferentiation rate of 15.83% was observed when 0.15 mg/L NAA + 0.20 mg/L 6-BA was used. The embryogenic callus redifferentiated into globular embryos (Figure 4b), and after 7 days, heart-shaped embryos and torpedo-shaped embryos gradually formed (Figure 4c). After 14 days, cotyledon-shaped embryos were formed (Figure 4d), which gradually turned white and matured into mature cotyledons. Upon transferring to light conditions, they greened (Figure 4e), and after inducing shoot germination and root formation, complete regenerated plants were obtained (Figure 4f). Plantlets regenerated from these embryos displayed normal phenotypes, including well-developed roots and shoots.

### 2.4. Cytological Observation

**Figure 5:**
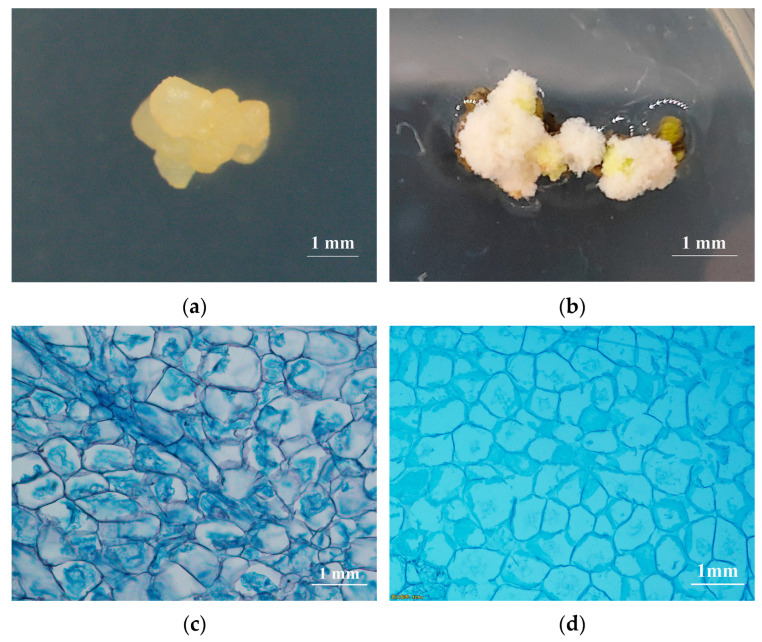
Phenotypic and cytological observation of two types of callus: The phenotype of embryogenic (**a**) and non-embryogenic (**b**) callus. Cytological observations of embryogenic (**c**) and non-embryogenic (**d**) callus.

After transdifferentiation of the explants, two types of callus were formed. One type was yellowish-white transparent callus (Figure 5a), which was relatively firm, with smooth, globular particles on the surface, making it easy to peel off. The other type was white callus (Figure 5b), which was loose in texture with a rough surface. Cytological observation of both types of callus through paraffin sectioning revealed significant differences. The first type of callus stained more easily, with smaller cells rich in inclusions, and the cell nuclei were clearly visible (Figure 5c). This type of callus gradually underwent SE in subsequent subcultures and was identified as embryogenic callus. The second type of callus stained more lightly, with larger cells containing fewer inclusions and no obvious cell nuclei (Figure 5d). This type of callus gradually browned and died in subsequent subcultures and was identified as non-embryogenic callus.

### 2.5. Transcriptomic Analysis at the Early Stage of Callus Redifferentiation

#### 2.5.1. Analysis and Functional Enrichment of DEGs During the Early Stage of Callus Redifferentiation

**Figure 6:**
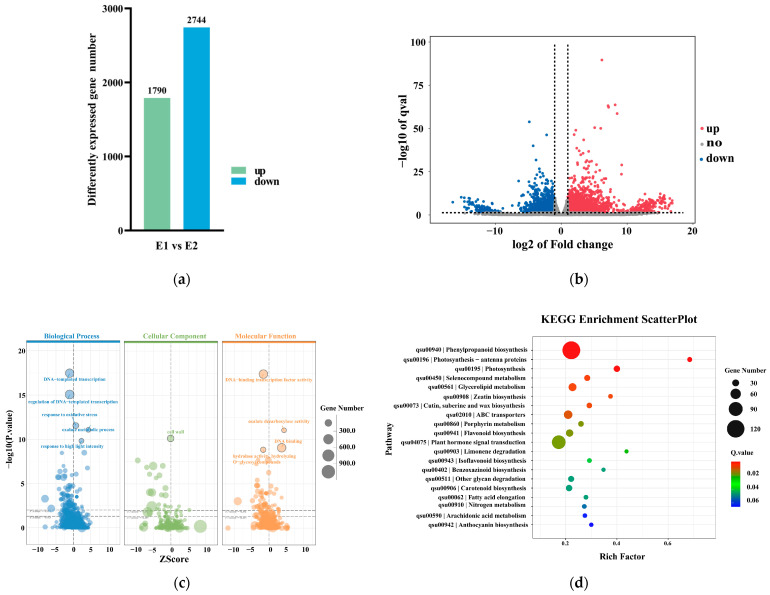
Analysis and Functional Enrichment of DEGs during the early stage of callus redifferentiation: (**a**) Numbers of up and down-regulated DEGs; (**b**) Volcano plot of DEGs (Red dots: significantly upregulated genes, Green dots: significantly downregulated genes, Gray dots: non-differentially expressed genes). (**c**) GO enrichment analyses of DEGs; (**d**) KEGG enrichment analyses of DEGs.

To gain a deeper understanding of the molecular mechanisms of the early stage of callus redifferentiation, we analyzed the DEGs during the early stage of callus redifferentiation (from E1 to E2). E1 samples were derived from proliferating embryogenic callus cultured on Q2 medium (MSSH liquid medium supplemented with 0.50 mg/L 6-BA and 0.20 mg/L NAA). E2 samples were obtained from globular embryos cultured on Q3 medium (MSSH solid medium supplemented with 0.15 mg/L NAA and 0.20 mg/L 6-BA). These samples represent distinct stages of early differentiation, with E1 corresponding to the early proliferative stage of callus and E2 corresponding to the early differentiation stage of somatic embryos. The results showed that 4534 genes were involved in the early stage of callus redifferentiation, with 1790 genes significantly upregulated and 2744 genes significantly downregulated (Figure 6a,b).

Gene Ontology (GO) enrichment analysis of the DEGs revealed the following results (Figure 6c). In the biological process category, DEGs were mainly enriched in “DNA-templated transcription”, “regulation of DNA-templated transcription”, “response to oxidative stress”, “oxalate metabolic process”, and “response to high light intensity”. A significant number of DEGs were also enriched in the “cell wall” category within cellular components. In the molecular function category, DEGs were predominantly enriched in “DNA-binding transcription factor activity”, “oxalate decarboxylase activity”, “DNA binding”, and “hydrolase activity, hydrolyzing O-glycosyl compounds”. These results indicate that the early stage of callus redifferentiation is primarily regulated by DNA transcription and cell wall development. Biological processes such as oxalate metabolism, oxidative stress response, and high light intensity response were also enriched, suggesting the early stage of callus redifferentiation may be regulated by environmental stress, metabolic processes, and signal transduction.

Kyoto Encyclopedia of Genes and Genomes (KEGG) enrichment analysis of the DEGs revealed that they were involved in 129 metabolic pathways, with a strong enrichment in the following pathways: “phenylpropanoid biosynthesis”, “photosynthesis—antenna proteins”, “photosynthesis”, “selenocompound metabolism”, “glycerolipid metabolism”, “cutin, suberin and wax biosynthesis”, “zeatin biosynthesis”, and “ABC transporters” (Figure 6d).

#### 2.5.2. Key Genes and TFs Associated with the Early Stage of Callus Redifferentiation and Their Functional Analysis

**Figure 7:**
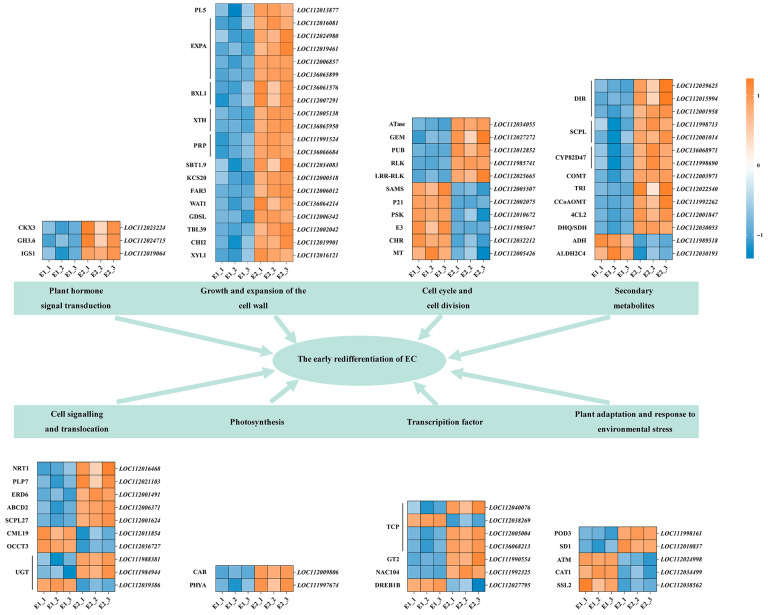
Relative expression patterns of the DEGs involved in the early stage of callus redifferentiation.

Based on functional annotation, we identified the top 100 key genes closely associated with early callus redifferentiation. We found that these genes are involved in hormone signal transduction, cell wall growth and expansion, cell proliferation and cell cycle regulation, secondary metabolism, signal transduction and transport, photosynthesis, TFs, and the adaptation and regulation of plants under environmental stress (Figure 7).

A group of genes related to plant hormone signaling pathways and cell wall remodeling were significantly upregulated during the E1 to E2 transition. Specifically, cytokinin dehydrogenase 3 (*CKX3*), indole-3-acetic acid-amido synthetase gene (*GH3.6*), and isoeugenol synthase 1 (*IGS1*)—key regulators of cytokinin and auxin homeostasis—exhibited increased expression by 1379-fold, 14-fold, and 3-fold at the E2 stage, suggesting their roles in promoting cellular redifferentiation. Simultaneously, genes involved in cell wall loosening and restructuring, including pectate lyase 5 (*PL5*), expansin A (*EXPA*), xyloglucan endotransglucosylase/hydrolase protein (*XTH*), and repetitive proline-rich cell wall protein (*PRP*), were upregulated, reflecting enhanced cell extensibility and structural adaptation during early somatic embryo formation.

In terms of cell cycle activation and secondary metabolism, several genes crucial for cell division and meristematic activity—such as S-adenosylmethionine synthase (*SAMS*), phytosulfokines (*PSK*), protein P21 (*P21*), and E3 ubiquitin–protein ligase (*E3*)—were more highly expressed at the E2 stage. These likely support renewed cell proliferation as embryonic structures emerge. Concurrently, genes involved in phenylpropanoid biosynthesis and lignin-related pathways (e.g., caffeic acid 3-O-methyltransferase (*COMT*), cytochrome P450 (*CYP82D47*), 4-coumarate—CoA ligase 2 (*4CL2*), and alcohol dehydrogenase (*ADH*)) were differentially upregulated, suggesting metabolic reinforcement to support differentiation and defense.

We also observed pronounced changes in stress-responsive and transport-associated genes. Redifferentiation appears to trigger signaling events and physiological adaptation, as indicated by the induction of cationic amino acid transporter 1 (*CAT1*), peroxidase 3 (*POD3*), protein strictosidine synthase-like 2 (*SSL2*), and receptor-like serine/threonine–protein kinase (*SD1*), which are associated with oxidative stress regulation and detoxification. Transporters including nitrate transporter (*NRT1*), sugar transporter (*ERD6*), organic cation/carnitine transporter (*OCCT3*), and the calcium sensor putative calcium-binding protein (*CML19*) were also induced, suggesting enhanced intracellular signaling, nutrient redistribution, and hormone translocation in support of morphogenic progression.

Finally, several genes involved in photosynthesis and transcriptional regulation were differentially expressed. Despite being in an early developmental stage, light-harvesting complex genes such as chlorophyll a-b binding protein (*CAB*) and phytochrome A (*PHY-A*) were mildly upregulated, possibly reflecting chloroplast biogenesis priming. Notably, transcription factors including TCP (*TCP4* and *TCP13*), NAC domain-containing protein 104 (*NAC104*), Trihelix (*GT2*), and dehydration responsive element-binding protein (*DREB1B*) exhibited strong expression changes, suggesting their central roles in orchestrating gene regulatory networks that govern cell fate transitions and embryogenic competence acquisition.

These results indicate that these DEGs play important roles in the early redifferentiation of callus.

#### 2.5.3. Selection of Reference Genes and Quantitative Real-Time PCR (qRT-PCR) Validation of DEGs

To ensure accurate normalization in qRT-PCR analysis, five commonly used candidate reference genes—*Actin*, RNA polymerase II (*QsRPII*), β-tubulin (*QsTUB*), Eukaryotic translation initiation factor 5A (*QsEIF-5A*), and Clathrin adaptor complexes medium subunit family protein ((*QsCACs*)*^C^*)—were evaluated for expression stability across two developmental stages, E1 and E2. The mean cycle threshold (Ct) values and corresponding standard deviations (SDs) were calculated based on three biological replicates for each stage (Table 2).

In addition, this candidate reference genes were evaluated using GeNorm, NormFinder, and BestKeeper algorithms based on Ct values from six biological samples, each with triplicate measurements (Table 3).

The GeNorm algorithm ranked candidate genes based on average pairwise variation (M value). All five genes exhibited acceptable stability (M < 1.5), with *QsEIF-5A* (M = 0.067) and *QsTUB* (M = 0.068) showing the highest stability, while *QsRPII* had the highest M value (0.101), indicating lower stability. NormFinder, which accounts for both intra- and inter-group variation, identified *QsTUB* as the most stable gene (variance = 0.0239), closely followed by *QsEIF-5A* (variance = 0.0309). *QsRPII* again showed the least stability (variance = 1.8197). BestKeeper ranks genes by SD of raw Ct values. Genes with SD > 1.0 are generally considered unstable. *QsEIF-5A* had the lowest SD (0.2236), followed by *QsTUB* (0.2707), whereas *QsRPII* (SD = 2.1981) and (*QsCACs*)*^C^* (SD = 1.7609) exhibited high variability.

Among the tested genes, *QsEIF-5A* exhibited the lowest variation in Ct values across the two stages, with average Ct values of 19.74 (SD = 0.19) in E1 and 19.59 (SD = 0.17) in E2, indicating high expression stability. *QsTUB* also demonstrated low variation (SD < 0.35), while *QsRPII* and *Actin* showed moderate variation. In contrast, (*QsCACs*)*^C^* presented the highest average Ct values (above 33) and moderate SDs, suggesting lower expression consistency under the tested conditions.

Based on analysis of all three methods, overall expression stability and Ct variation, *QsEIF-5A* were ranked as the most stable reference genes under the tested conditions, and were therefore selected for normalization in subsequent qRT-PCR expression analysis.

To verify the reliability of the RNA-seq data, six DEGs were selected for validation by qRT-PCR. These genes included two hormone-signal-transduction-related genes, one gene associated with cell wall growth and expansion, one involved in secondary metabolism, one related to adaptation and regulation of plants under environmental stress, and one transcription factor.

**Figure 8:**
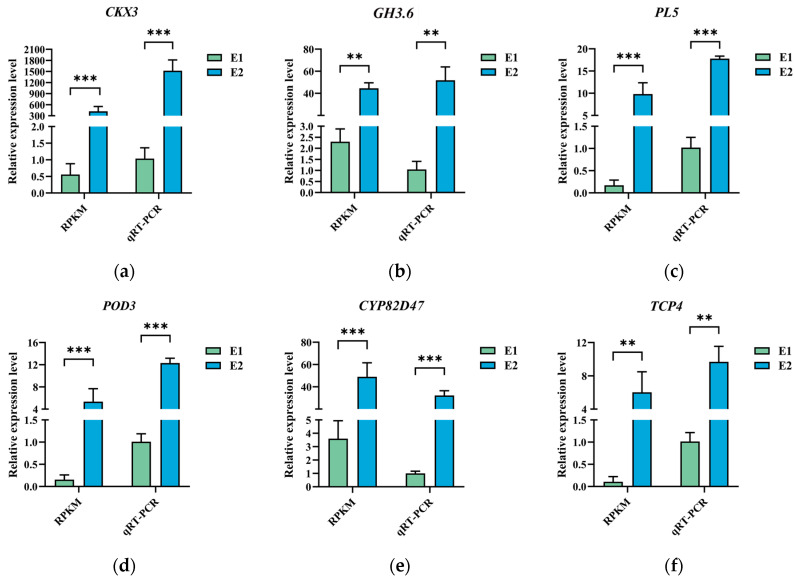
Relative expression level of *CKX3* (**a**), *GH3.6* (**b**), *PL5* (**c**), *POD3* (**d**), *CYP82D47* (**e**), and *TCP4* (**f**), while the corresponding RPKM values from RNA-seq are shown for comparison (*N* = 3, *R* = 3, All data are shown as mean ± SD. ** *p* < 0.05, *** *p* < 0.001).

The qRT-PCR results showed high consistency with the RNA-seq data, as the expression patterns at developmental stages E1 and E2 closely matched the Reads Per Kilobase of transcript per Million mapped reads (RPKM) values from the RNA-seq analysis (Figure 8). Furthermore, a strong positive correlation was observed between the relative expression levels of the DEGs (based on 2^−ΔΔCt^ qRT-PCR values) and their corresponding RPKM values from RNA-seq (*p* < 0.05).

These results collectively confirm the high reliability and accuracy of the RNA-seq dataset.

## 3. Discussion

### 3.1. Transdifferentiation to Form Callus of Q. suber

Differentiated cells returning to an earlier developmental state under certain environmental conditions is referred to as transdifferentiation [50]. In plants, callus is widely regarded as a proliferating mass of transdifferentiation cells [14]. Transdifferentiation is the first step in plant SE, during which the developmental potential of cells is enhanced, and this process is influenced by various factors. Explants for inducing somatic embryos have diverse sources, and immature cotyledons are commonly used as explants for induction [51]. In recent years, induction has also been performed using organs such as leaves, stems, and roots [52]. Liang et al. [53] found that petiole-derived explants produced more somatic embryos than leaf mesophyll, which is consistent with the results of this study. We hypothesize that the petiole, as a vascular tissue involved in nutrient transport, likely requires more structural and organizational support to ensure sufficient mechanical support and transportation functions, thus producing more somatic embryos than mesophyll.

The time of sampling not only affects contamination rates but also influences the dedifferentiation rate. Testillano et al. [26] discovered that immature zygotic embryos are more likely to form somatic embryos compared to mature zygotic embryos. In this study, explants collected in May showed a significantly higher transdifferentiation rate than those collected at other times. We speculate that May is the peak growing season for plants, with more active cell differentiation, which likely enhances the ability of cells to transdifferentiate and return to an undifferentiated state. During dormancy or maturation periods, plant cell differentiation tends to be more stable, and the transdifferentiation capacity may decrease.

The medium generally consists of a basal medium and PGRs, both of which play a significant role in the transdifferentiation process [54]. Additionally, different plants require different light conditions for transdifferentiation. Pinto et al. [30] found that *Q. suber* from 60-year-old trees cultured in the dark on MS + 0.99 mg/L 2,4-D + 1.97 mg/L ZT medium were more conducive to transdifferentiation. In this study, we found that dark conditions were more favorable for transdifferentiation, and we optimized the medium and plant growth regulator combinations, achieving a transdifferentiation rate of 67.62%. This experiment established a more efficient transdifferentiation protocol, which will facilitate the acquisition of more embryogenic callus for subsequent experiments.

### 3.2. Callus Proliferation

After transdifferentiation, the callus generally requires subculture, which not only promotes the proliferation of the callus but also supports the subsequent formation of embryogenic callus [55]. Liquid culture is commonly chosen as a subculture method. Zhai et al. [56] found that starting with 2.00 g of callus for liquid culture was more beneficial for increasing cell biomass. In our study, inoculating 1.00 g of callus into liquid culture proved to be more effective for enhancing proliferation, whereas higher inoculum levels (3.00 g) resulted in reduced proliferation and slight browning.

It should be noted, however, that only two mass-to-volume ratios (1 g and 3 g per 9 mL medium) were tested, which provides a relatively narrow range for evaluating the full potential of liquid culture. Future studies should include a broader spectrum of inoculum sizes and medium volumes to further optimize culture conditions for large-scale applications. Moreover, although our results clearly demonstrate the advantage of liquid culture for proliferation, cytological observations of callus morphology and recovery assays after transferring callus back to solid medium were not conducted. Such analyses are important to ensure that embryogenic competence is maintained and that liquid culture does not induce dramatic cellular changes. Previous studies have emphasized the importance of cytological validation and recovery testing in confirming the developmental stability of embryogenic callus under different culture systems [57,58]. Incorporating these approaches in future research will strengthen our understanding of liquid culture systems.

Currently, liquid culture for *Q. suber* remains in the exploratory stage. The present study provides scientific evidence for improving the SE system of *Q. suber* and contributes to its future industrial application.

### 3.3. Somatic Embryo Induction Embryo, Maturation and Germination

The transformation of embryogenic callus into a complete plant involves several important stages. The first stage is the transition of embryogenic callus into globular embryos (the early stage of callus redifferentiation); the second stage involves the development of global embryo into heart-shape embryo, torpedo-shape embryo, and Cytoledon-stage embryo; the third stage involves the induction of Cytoledon-stage embryos into maturation and germination to form a complete plant [59]. The redifferentiation stage is mainly influenced by PGRs. Pinto et al. [30] observed SE in leaf-derived callus only on MS + 0.99 mg/L 2,4-D + 1.97 mg/L ZT medium. Fernández-Guijarro et al. [60] found that low concentrations of 6-BA + NAA were beneficial for initiating the early stage of callus redifferentiation, which is consistent with the results of this study. Although quantitative data on embryo number per gram of callus were not collected, our morphological observations confirmed the frequent initiation of somatic embryos. Similar observations have been reported [61]. Plantlets regenerated from these embryos displayed normal phenotypes, including well-developed roots and shoots, consistent with previous studies. This experiment optimized the redifferentiation protocol, providing new insights into the redifferentiation process of *Q. suber*.

### 3.4. Transcriptomics

The early stage of callus redifferentiation, as a crucial stage in SE, has been successively reported in various plants in recent years [62]. The regenerative ability of embryogenic callus is controlled by multiple genes and has been extensively studied in many plant species [63].

The metabolism of plant hormones is essential during the cell differentiation stage. Avilez-Montalvo et al. [64] found that *CKX3*, which encodes cytokinin oxidase, begins to express at the start of induction and increases sharply until day 21 of SE induction when the first globular structure appears, which is consistent with the results of this study. In this study, 0.50 mg/L 6-BA during the early redifferentiation period effectively increased the redifferentiation rate, suggesting that 6-BA is the primary cytokinin that regulates the redifferentiation of *Q. suber*, potentially regulated by *CKX3*. *GH3* encodes an enzyme that catalyzes the esterification of indole-3-acetic acid (IAA) to various amino acids. Méndez-Hernández et al. [65] found that *GH3.5* is only expressed during the first few days of embryogenesis and decreases during the induction phase, which is consistent with our findings. *GH3.5* is homologous to *AtGH3.11*, and we hypothesize that *GH3.6* may be involved in the binding of jasmonic acid (JA) and isoleucine.

The growth and extension of the cell wall play a crucial role in the morphological changes and early redifferentiation of callus [66], and this effect has also been confirmed in *Q. suber* [67]. In this study, it was found that the expression levels of *PLs* and important genes related to cell wall loosening and expansion (such as *EXPs* and *XTHs*) were significantly higher during the induction process compared to the E1 stage, indicating that they play an important role in the early redifferentiation process.

Genes related to cell proliferation and the cell cycle also play a key role in regulating early redifferentiation. *SAMS* plays an important role in the biosynthesis of polyamines and ethylene [68]. Studies have shown that both free and bound spermine inhibit embryogenic potential, with the highest *SAMS* activity and spermine levels correlating with the lowest embryogenic cell levels [69], which aligns with the results of this study.

Secondary metabolism is considered an important mechanism for plant adaptation to ecological environments, and it plays a role in cellular activity and plant growth and development. Signal transduction and transport accompany plant growth throughout its life cycle. Cytochrome P450, as a key enzyme in drug metabolism, plays a significant role not only in cytokine activity and thermoregulation but also in early SE in longan [70], which is consistent with our findings.

Key genes and transcription factors regulate processes such as cell dedifferentiation, formation of embryogenic cells, and embryo development. SE is influenced not only by endogenous genes but also by external environmental factors. Stress responses and environmental adaptation play a vital role in the induction of SE, while photosynthesis is essential for plant growth and development at all stages. *CAB*, a component of the PSII light-harvesting complex, was significantly upregulated in this study, further confirming its key role in SE. In our experiment, dark conditions were more favorable for early redifferentiation of embryogenic callus, and we hypothesize that this process is primarily regulated by *CAB*. The *TCP* gene family was upregulated during somatic embryogenesis, regulating cell proliferation and differentiation to protect the embryo [71], which is consistent with our results. Additionally, *POD3* plays an important role in the formation of the embryonic wall and has been proposed as a marker for SE [72]. The significant upregulation of *POD3* in this experiment further supports its crucial role in SE.

In transcriptomic studies, normalization methods such as RPKM and Transcripts Per Million (TPM) are commonly used to adjust for differences in sequencing depth and gene length [73]. Both methods aim to provide comparable expression values across samples, but they differ in their normalization strategies. RPKM normalizes for gene length and sequencing depth by scaling the read count of each gene to the total number of reads, while TPM first normalizes for sequencing depth by scaling the total number of reads per sample to a fixed value (typically one million) and then normalizes for gene length. While TPM is generally considered the more robust method, particularly for comparing gene expression across samples with varying library sizes, RPKM has been widely used in many studies and can offer similar results in many cases [74]. In fact, the difference between RPKM and TPM is often small, especially when the read count distributions are relatively uniform across samples [75,76]. Nonetheless, TPM is preferred when there is a need to compare gene expression levels between multiple samples within a dataset, as it avoids the issue of bias introduced by differences in sequencing depth. In this study, we chose to use RPKM values. We recognize the advantages of using TPM for future studies and will consider it for future analyses where sample comparisons across conditions or datasets are critical.

Among the top 100 DEGs, 12 genes lacked functional annotations, 12% lacked functional annotations. While these uncharacterized genes may represent novel regulators of somatic embryogenesis, the majority of annotated genes were significantly enriched in pathways related to transcription regulation, cell wall development, stress responses, thereby supporting the reliability of our conclusions. The 12 genes lacked functional annotations including 10 predicted long non-coding RNAs (lncRNAs). Although their precise functions remain unclear, lncRNAs are known to act as important regulators in plant development and stress adaptation [77,78], suggesting that these unannotated genes may also contribute to the regulation of early callus redifferentiation.

In summary, these findings suggest that the early stage of callus redifferentiation involves coordinated regulation by multiple genes, metabolic pathways, and environmental factors. This study has preliminarily identified key candidate TFs for early callus redifferentiation, providing a foundation for further research on the molecular mechanisms of SE in *Q. suber*.

## 4. Materials and Methods

### 4.1. Plant Material

Immature leaves were collected in 2023 from two-year-old *Q. suber* seedlings grown at the Research Institute of Non-Timber Forestry, Chinese Academy of Forestry, Zhengzhou, Henan, China (113°41′57″ N, 34°46′19″ E). The seedlings originated from seeds obtained from the Xiaogan City Materials Institute. Young apical leaves were selected for somatic embryo induction. Prior to inoculation, the leaves were brushed with a soft brush to remove surface trichomes, rinsed under running water for 30 min, immersed in 75% (*v*/*v*) ethanol for 10 s, and then surface-sterilized with 1% NaClO for 5 min. Sterile water washes (3–5 times) were performed after both ethanol and NaClO treatments, and the material was dried on sterile filter paper. For the experiments, each leaf was divided into three explant types (petiole, mesophyll, and leaf vein), which were subsequently inoculated onto culture media for callus induction and somatic embryogenesis. Living plantlets regenerated from these cultures are maintained at the Research Institute of Non-Timber Forestry (Zhengzhou, China).

### 4.2. Medium and Culture Conditions

All media used in the experiment had their pH adjusted to 5.8 ± 0.1 using NaOH or HCl, followed by autoclaving at 121 °C for 20 min.

#### 4.2.1. Callus Induction

The explants were inoculated onto the Q1 culture medium and incubated in the dark at 25 °C for 30 days. The Q1 medium consisted of the MSSH basic medium supplemented with 0.50 mg/L 6-BA, 1.00 mg/L NAA, 30 g/L sucrose, and 7 g/L agar.

#### 4.2.2. Callus Proliferation

Embryogenic callus, with consistent growth and developmental stages, weighing either 1.00 g, was inoculated into the 9 mL Q2 medium and cultured at 100 r/min in the dark. Fresh medium was transferred every week. The Q2 medium consisted of MSSH basic medium supplemented with 0.20 mg/L 6-BA, 0.50 mg/L NAA, and 30 g/L sucrose.

#### 4.2.3. Somatic Embryo Induction

Embryogenic callus with consistent growth and developmental stages was inoculated onto the Q3 medium and incubated in the dark at 25 °C for 30 days. The Q3 medium consisted of MSSH basic medium, supplemented with 0.15 mg/L NAA, 0.20 mg/L 6-BA, 30 g/L sucrose, and 7 g/L agar.

#### 4.2.4. Embryo Maturation and Germination

To stimulate maturation, once the somatic embryos reached the cytoledon-stage, they were transferred to the best hormone-free medium using the method by Vladimír Chalupa [33], with a light cycle of 16 h light and 8 h dark, until the cytoledon-stage embryos turned green. To induce germination, the green cytoledon-stage embryos were then transferred to MSSH medium supplemented with 0.50 mg/L 6-BA and 0.10 mg/L NAA and cultured until shoot formation with at least two leaves. Subsequently, the shoots were transferred to MSSH medium containing 1.2 mg/L indole-3-butyric acid (IBA) and 0.05 mg/L NAA until root formation.

### 4.3. Various Factors Effect on Transdifferentiation to Form Callus

All explants were inoculated onto the culture medium after different treatments and incubated in the dark at 25 °C for 30 days. The growth of the explants was observed and recorded regularly, and the transformation rate was determined after 30 days. Each experimental group contained 45 samples, with 3 repetitions per group.

Transdifferentiation rate = (Number of explants with callus formation/Total number of inoculated explants) × 100%(1)

#### 4.3.1. Explant Types Effect on Transdifferentiation to Form Callus

For the experiments, each leaf was divided into three explant types (petiole, mesophyll, and leaf vein), and inoculated onto the Q1 culture medium and incubated in the dark. The most suitable explant type was selected for subsequent experiments.

#### 4.3.2. Sampling Time Effect on Transdifferentiation to Form Callus

Explants collected on 10 May, 10 August, 10 November, and 10 January of the following year were inoculated onto the Q1 medium and incubated in the dark. The most suitable sampling time was selected for subsequent experiments.

#### 4.3.3. Type of Basic Medium Effect on Transdifferentiation to Form Callus

Explants were inoculated onto the Q4 medium and incubated in the dark. The Q4 medium included MS basic medium, SH basic medium, or MSSH basic medium, supplemented with 0.50 mg/L 6-BA, 1.00 mg/L NAA, 30 g/L sucrose, and 7 g/L agar. The most suitable type of basic culture medium was selected for subsequent experiments.

#### 4.3.4. Light Conditions Effect on Transdifferentiation to Form Callus

Explants were inoculated onto the Q1 medium and incubated under light conditions (16 h light, 8 h dark, light intensity 2000 lx–3000 lx) or in the dark (24 h dark). The most suitable light condition was selected for subsequent experiments.

#### 4.3.5. PGRs on Transdifferentiation to Form Callus

Explants were inoculated onto the Q5 medium and incubated in the dark. The Q5 medium consisted of MSSH basic medium, supplemented with NAA (0.00 mg/L, 1.00 mg/L, 2.00 mg/L) and 6-BA (0.00 mg/L, 1.50 mg/L, 3.00 mg/L), 30 g/L sucrose, and 7 g/L agar. The most suitable PGRs combination was selected for subsequent experiments.

### 4.4. Various Factors Effect on Proliferation

All explants were inoculated onto the culture medium after different treatments and incubated in the dark at 25 °C for 30 days. The growth of the callus was observed regularly, and after 30 days, the quality and proliferation rate of the embryogenic callus were recorded. There were 3 replicates in each group.

Proliferation rate = (Final embryogenic callus weight − Initial inoculated embryogenic callus weight)/Initial inoculated embryogenic callus weight(2)

#### 4.4.1. PGRs

Embryogenic callus, with consistent growth and developmental stages were inoculated onto the Q6 medium and incubated in the dark at 25 °C for 30 days. The Q6 medium consisted of MSSH basic medium, supplemented with NAA (0.10 mg/L, 0.15 mg/L, 0.20 mg/L, 0.25 mg/L) and 6-BA (0.10 mg/L, 0.50 mg/L, 0.80 mg/L, 1.00 mg/L), 30 g/L sucrose, and 7 g/L agar.

#### 4.4.2. Culture Method

Embryogenic callus, with consistent growth and developmental stages, weighing either 1.00 g or 3.00 g, was inoculated into the Q2 medium (Callus weight:medium volume = 1:9) and cultured at 100 r/min in the dark, designated as fluid1 and fluid2. Fresh medium was transferred every week. Additionally, 1.00 g of healthy, stage-consistent embryogenic callus was inoculated onto the Q7 medium at 0 r/min in the dark, designated as solid1. The Q7 medium consisted of Q2 medium supplemented with 7 g/L agar.

### 4.5. PGRs Effect on Somatic Embryo Induction

Embryogenic callus with consistent growth and developmental stages was inoculated onto the Q7 medium and incubated in the dark at 25 °C for 30 days. The Q7 medium consisted of MSSH basic medium, supplemented with NAA (0.10 mg/L, 0.15 mg/L, 0.20 mg/L, 0.25 mg/L) and 6-BA (0.20 mg/L, 0.30 mg/L, 0.40 mg/L), 30 g/L sucrose, and 7 g/L agar. All explants were inoculated onto the culture medium after different treatments and incubated at 25 °C for 30 days. The growth of the explants was observed and recorded regularly, and the transformation rate was determined after 30 days. Each experimental group contained 45 samples, with 3 repetitions per group. Embryogenic callus developed embryoids that progressed sequentially through the global, heart-shape, torpedo-shape, and cotyledon-shape stages. Although multiple embryoids were typically formed per callus mass, no systematic quantification was performed in this study.

Redifferentiation rate = (Number of embryogenic callus showing embryoids/Total number of inoculated embryogenic callus) × 100%(3)

### 4.6. Cytological Observation

Different types of embryogenic callus with consistent growth and developmental stages were selected for cytological observation using the method of Ok Ran Lee et al. [79]. After fixation, dehydration, and clearing, the samples were immersed in LR White resin (London Resin Co., London, UK). The samples were then sectioned to a thickness of 9 μm and stained with 0.05% toluidine blue. Observations were made under a light microscope (Zeiss, Axiolab, Jena, Germany). Images were captured using Slide Viewer 2.5 software [80]. Each experimental group contained 3 samples, with at least five fields of view selected for each sample.

### 4.7. Transcriptome Sequencing and DEGs Analysis

Total RNA was extracted from E1 and E2 using the QIAGEN RNeasy Plant Standard Kit (QIAGEN, Hilden, Germany), following the kit’s instructions. Three biological replicates were set for each stage, and each sample weighed at least 0.50 g. The samples were stored at ultra-low temperatures after collection. The RNA integrity and concentration were assessed using 1% agarose gel electrophoresis and an Agilent 2100 Bioanalyzer (Agilent Technologies, Palo Alto, CA, USA). The RNA libraries were sequenced on the illumina NovaseqTM 6000 platform by LC Bio Technology CO., Ltd. (Hangzhou, China). The raw sequences were filtered using FastQC 0.10.1 software [81] to remove adapter sequences, reads with more than 5% unknown nucleotides (N), and low-quality reads (with more than 40% of bases having a quality value Q ≤ 25). High-quality clean reads were obtained. Hisat2 2.2.1 software was used to map the final clean reads to the European cork oak reference genome (https://ftp.ncbi.nlm.nih.gov/genomes/all/GCF/002/906/115/GCF_002906115.3_Cork_oak_2.0, accessed on 24 September 2024). Transcripts were assembled to generate gene sequences. DEGs were identified using DESeq2 1.22.2 software [82], with the criteria set as |fold change| > 1 and false discovery rate (FDR) < 0.05. Furthermore, DEGs were detailed according to GO functions and KEGG pathways.

### 4.8. Selection of Reference Genes and Gene Expression Validation via qRT-PCR

Total RNA was extracted using the RNE36 DNase I (RNase-Free) Kit (Nobelab Biotech. Co., Ltd., Nanjing, China) following the manufacturer’s protocol. First-strand complementary DNA (cDNA) was synthesized from purified RNA using the ReScript II RT All-in-One Mix (with dsDNase) Kit (Nobelab Biotech. Co., Ltd., Nanjing, China), in accordance with the supplier’s instructions.

Specific primers for five reference genes (selected based on published literature) [83] and six target genes were designed using Primer Premier 5.0 software [84]. The detailed primer sequences are listed in Table A1.

The qRT-PCR reactions were conducted using the 2× SYBR Premix UrTaq II Kit (Nobelab Biotech. Co., Ltd., Nanjing, China) on a CFX384 Touch Real-Time PCR Detection System (Bio-Rad Laboratories, Inc., Hercules, CA, USA). Each reaction included three biological replicates per sample. Relative gene expression levels were calculated using the 2^−ΔΔCt^ method [85].

### 4.9. Data Analysis

All experimental data were organized using Microsoft Excel 2010 [86]. Statistical analysis was performed using SPSS 26.0 software [87] for one-way analysis of variance (ANOVA), with significant differences verified by Duncan’s multiple range test, and a significance level set at *p* < 0.05. Graphs were generated using GraphPad Prism 9.5 [88], Origin 64 [89] and R 3.6 software [90] based on the analysis results. The development stages of zygotes and somatic embryos were observed using a somatic cell microscope (Leica S8AP0, Leica Microsystems, Heerbrugg, SG, Switzerland), while the microscopic structure was observed using a Zeiss inverted microscope (ZEISS DMI4000, Carl Zeiss AG, Oberkochen, BW, Germany).

## 5. Conclusions

This study establishes an efficient SE pipeline for *Q. suber* by optimizing culture conditions and profiling the early redifferentiation transcriptome. Petioles and leaf veins were identified as the most responsive explants, with May as the optimal sampling time. Among basal media and light regimes, MSSH medium under darkness maximized transdifferentiation, and the PGRs combination of 0.50 mg/L 6-BA + 1.00 mg/L NAA yielded the highest callus induction. For proliferation, liquid culture with a 1.00 g inoculum outperformed solid culture, and 0.50 mg/L 6-BA + 0.20 mg/L NAA was optimal. Redifferentiation of embryogenic callus reached 15.83% with 0.15 mg/L NAA + 0.20 mg/L 6-BA, producing morphologically normal plantlets after maturation and germination. Cytology distinguished compact, inclusion-rich embryogenic callus from loose, low-staining non-embryogenic callus.

Transcriptome analysis of the early redifferentiation stage (E1 → E2) revealed 4534 DEGs and enrichment of processes related to DNA-templated transcription, cell-wall remodeling, oxidative stress, oxalate metabolism, and light response. KEGG terms were dominated by phenylpropanoid and lipid-derived pathways, photosynthesis, cutin/suberin/wax biosynthesis, zeatin biosynthesis, and ABC transporters. Key regulators included *CKX3*, *GH3.6*, *IGS1*, *PL5*, *CYP82D47*, *POD3* and TFs (*TCP4*). qRT-PCR validation (normalized to *QsEIF-5A*) confirmed RNA-seq trends (*p* < 0.01).

Together, these findings define practical parameters for in vitro regeneration of *Q. suber* and provide molecular leads for dissecting early callus redifferentiation.

## Figures and Tables

**Table 1:** Effects of sampling time on transdifferentiation of *Q. suber*.

Sampling Time	Transdifferentiation Rate (%)	Callus Growth Status
10 May	62.22 ± 4.45 a	The callus appeared yellowish-white, with a large quantity and vigorous growth.
10 September	38.52 ± 6.78 b	The explants were light purple in color, with the callus growing vigorously and showing slight browning.
10 November	27.41 ± 5.59 c	The explants were purple-black in color, with the callus mostly white and in small quantities.
10 January of the following year	7.41 ± 2.57 d	The explants exhibited severe browning, turning blackish-purple in color, and the callus formed in small quantities.

Note: Means ± SD error within a column. Different letters meant there was significant difference among groups, *p*  <  0.05. *N* = 45, *R* = 3.

**Table 2:** Mean Ct Values ($\bar{x}$ ± SD) of Five Candidate Reference Genes during the Early Stage of Callus Redifferentiation in *Q. suber*.

The Early Stage of Callus Redifferentiation	Genes				
	*Actin*	*QsRPII*	*QsTUB*	*QsEIF-5A*	*Qs(CACs)^C^*
E1	29.27 ± 1.40	31.89 ± 2.50	32.61 ± 0.34	19.74 ± 0.19	33.19 ± 1.28
E2	28.43 ± 1.36	31.46 ± 2.84	32.46 ± 0.30	19.59 ± 0.17	33.52 ± 1.25

**Table 3:** Comparative Analysis of Reference Gene Stability Based on Three Algorithms.

Genes	Algorithm		
	GeNorm_M	NormFinder_Var	BestKeeper_SD
*Actin*	0.067090	0.030921	0.223572
*QsRPII*	0.068351	0.023950	0.270710
*QsTUB*	0.084357	0.798149	1.404111
*QsEIF-5A*	0.091297	0.499828	1.760916
*Qs(CACs)^C^*	0.101352	1.819655	2.198074

## Data Availability

The datasets generated and analyzed during the current study are available in the [NCBI] repository, [PRJNA1248735].

## Abbreviations

The following abbreviations are used in this manuscript:

SE	somatic embryogenesis
6-BA	6-benzyladenine
NAA	1-Naphthaleneacetic acid
2,4-D	2,4-Dichlorophenoxyacetic acid
ZT	zeatin
IAA	indole-3-acetic acid
IBA	indole-3-butyric Acid
MS	Murashige and Skoog
SH	Schenk and Hildebrandt
MSSH	MS major elements + SH minor elements and vitamins
DEGs	differentially expressed genes
E1	embryogenic callus
E2	globular embryos
GO enrichment	Gene Ontology enrichment
KEGG enrichment	Kyoto Encyclopedia of Genes and Genomes enrichment
qRT-PCR	quantitative real-timepolymerase chain reaction
PGRs	plant growth regulators
TFs	transcription factors
*LEC*	LEAFY COTYLEDON
*BBM*	BABY BOOM
*SERK*	Somatic embryogenesis receptor-like kinase
*CYP82D47*	cytochrome P450
*POD3*	peroxidase 3
*EXPA*	expansin A
*XTH*	xyloglucan endotransglucosylase/hydrolase protein
*PRP*	repetitive proline-rich cell wall protein
*SAMS*	S-adenosylmethionine synthase
*PSK*	phytosulfokines
*P21*	protein P21
*E3*	E3 ubiquitin-protein ligase
*COMT*	caffeic acid 3-O-methyltransferase
*4CL2*	4-coumarate—CoA ligase 2
*ADH*	alcohol dehydrogenase
*CAT1*	cationic amino acid transporter 1
*SSL2*	protein strictosidine synthase-like 2
*SD1*	receptor-like serine/threonine-protein kinase
*NRT1*	Nitrate transporter
*ERD6*	sugar transporter
*OCCT3*	organic cation/carnitine transporter
*CML19*	the calcium sensor putative calcium-binding protein
*CAB*	chlorophyll a-b binding protein
*PHY-A*	phytochrome A
*NCA104*	NAC domain-containing protein 104
*GT2*	Trihelix
*DREB1B*	dehydrationresponsiveelemen-tbindingprotein
*QsRPII*	RNA polymerase II
*QsTUB*	β-tubulin
*QsEIF-5A*	Eukaryotic translation initiation factor 5A
*(QsCACs)^C^*	Clathrin adaptor complexes medium subunit family protein

## Appendix A

**Table A1:** The sequences of 11 genes and primer pairs.

Gene	Primers	Amplicon Size (bp)	Temperature (°C)
*Actin*	Forward: 5′-TGGATTCTGGTGATGGTGTGAGTC-3′	162	62
	Reverse: 5′-CAATTTCCCGTTCAGCAGTAGTGG-3′		
*QsRPII*	Forward: 5′-GACATAGATCCCGTTACCCA-3′	168	54
	Reverse: 5′-TTTGATTGCACCAGTAGATTC-3′		
*QsTUB*	Forward: 5′-GCTCACTACCCCAAGCTTT-3′	187	58
	Reverse: 5′-GGAACCTCTGGAGGTTAAA-3′		
*QsEIF-5A*	Forward: 5′-GCCATGTCCGACGAGGAG-3′	86	62
	Reverse: 5′-CGGATGGTTCCGGCTTGC-3′		
*Qs(CACs)^C^*	Forward: 5′-TCTGGGAGAAGAGTGGCTACA-3′	175	57
	Reverse: 5′-GAGCCACCATTCAAATCCT-3′		
*CKX3*	Forward: 5′-GCAATGGCTCCTAATGGGGT-3′	87	61
	Reverse: 5′-TGGGGTTCCAGAGACAGTGA-3′		
*GH3.6*	Forward: 5′-AGACCATCCCAGGCCACTAT-3′	165	62
	Reverse: 5′-TCTCAAGGGGCCCAATTGAC-3′		
*PL5*	Forward: 5′-CAAGGGCATGCAGGTCACTA-3′	155	61
	Reverse: 5′-TGATGGTTGGGGAAGCACTC-3′		
*POD3*	Forward: 5′-CTTGCTCTCAGGTGCTCACA-3′	104	61
	Reverse: 5′-ggT CTAGAGCTGGGTCCTGA-3′		
*CYP82D47*	Forward: 5′-GGTTGGACTTGGGAGGCTAC-3′	107	60
	Reverse: 5′-ACCTGAAATTTTTGCGCGCT-3′		
*TCP4*	Forward: 5′-TGGGGGCTGAGATGACCATA-3′	84	60
	Reverse: 5′-ACCGAGTGTTGTCGATGCTT-3′		

## References

[1] Ramos M., Rocheta M., Carvalho L., Inácio V., Graça J., Morais-Cecilio L. (2013). Expression of DNA methyltransferases is involved in *Quercus suber* cork quality. Tree Genet. Genomes.

[2] Soler M., Serra O., Molinas M., García-Berthou E., Caritat A., Figueras M. (2008). Seasonal variation in transcript abundance in cork tissue analyzed by real time RT-PCR. Tree Physiol..

[3] Chaves I., Lin Y.C., Pinto-Ricardo C., Peer Y.V., Minguel C. (2014). miRNA profiling in leaf and cork tissues of *Quercus suber* reveals novel miRNAs and tissue-specific expression patterns. Tree Genet. Genomes.

[4] Prasetia D., Purusatama B.D., Kim J.H., Yang G.U., Jang J.H., Park S.Y., Lee S.H., Kim N.H. (2022). Quantitative Anatomical Characteristics of Virgin Cork in *Quercus variabilis* Grown in Korea. Forests.

[5] He S.A., Wu H.J. (1964). Introduction of Eucalyptus and European Cork Oak in Nanjing. For. Sci..

[6] She Y.G. (2000). Germination Test of European Cork Oak Seeds. Econ. For. Res..

[7] Peng J.G. (1986). European Cork Oak. Sichuan For. Sci. Technol..

[8] Wang M.X. (1963). Improvement of Cork Oak Species in China. For. Sci..

[9] Hae N.K., Hye Y.J., Myeong J.K., Inkyin K., Ha N.Y., Tae Y.L., Tai H.A., Su Y.W. (2017). Why does *Quercus suber* species decline in Mediterranean areas?. J. Asia-Pac. Biodivers..

[10] Guo H.H., Qi X.S., Li T.T., Fan Y.J., Huo H.Q., Yu Q.Y., Zeng F.C. (2022). Editorial: Molecular basis of asexual reproduction and its application in crops. Plant Sci..

[11] Vieitez A.M., Corredoira E., Martínez M.T., San-José M.C., Sánchez C., Valladares S., Vidal N., Ballester A. (2012). Application of biotechnological tools to *Quercus* improvement. Eur. J. For. Res..

[12] Finer J.J., Gamborg O.L., Phillips G.C. (2013). Direct Somatic Embryogenesis. Plant Cell, Tissue and Organ Culture, Springer Lab Manual.

[13] Guo H., Guo H., Zhang L., Fan Y., Wu J., Tang Z., Zhang Y., Fan Y., Zeng F. (2020). Dynamic Transcriptome Analysis Reveals Uncharacterized Complex Regulatory Pathway Underlying Genotype-Recalcitrant Somatic Embryogenesis Transdifferentiation in Cotton. Genes.

[14] Fehér A. (2019). Callus, Dedifferentiation, Totipotency, Somatic Embryogenesis: What These Terms Mean in the Era of Molecular Plant Biology?. Front. Plant Sci..

[15] Sugimoto K., Gordon S.P., Meyerowitz E.M. (2011). Regeneration in plants and animals: Dedifferentiation, transdifferentiation, or just differentiation?. Trends Cell Biol..

[16] Chadipiralla K., Gayathri P., Rajani V., Reddy P.V.B., Roychowdhury R., Choudhury S., Hasanuzzaman M., Srivastava S. (2020). Plant Tissue Culture and Crop Improvement. Sustainable Agriculture in the Era of Climate Change.

[17] Awada R., Campa C., Gibault E., Déchamp E., Georget F., Lepelley M., Abdallah C., Erban A., Martinez-Seidel F., Kopka J. (2019). Unravelling the Metabolic and Hormonal Machinery During Key Steps of Somatic Embryogenesis: A Case Study in Coffee. Int. J. Mol. Sci..

[18] Guan Y., Li S.G., Fan X.F., Su Z.H. (2016). Application of Somatic Embryogenesis in Woody Plants. Front. Plant Sci..

[19] Ramírez-Mosqueda M.A., Ramírez-Mosqueda M.A. (2022). Overview of Somatic Embryogenesis. Somatic Embryogenesis.

[20] Zimmerman J.L. (1993). Somatic Embryogenesis: A Model for Early Development in Higher Plants. Plant Cell.

[21] Sivanesan I., Nayeem S., Venkidasamy B., Kuppuraj S.P., RN C., Samynathan R. (2022). Genetic and epigenetic modes of the regulation of somatic embryogenesis: A review. Biol. Futur..

[22] Kumaravel M., Uma S., Backiyarani S., Saraswathi M.S. (2020). Proteomic analysis of somatic embryo development in *Musa* spp. cv. Grand Naine (AAA). Sci. Rep..

[23] Wang F.X., Shang G.D., Wu L.Y., Xu Z.G., Zhao X.Y., Wang W.J. (2020). Chromatin Accessibility Dynamics and a Hierarchical Transcriptional Regulatory Network Structure for Plant Somatic Embryogenesis. Dev. Cell.

[24] Pre A., Obert B. (2003). Flax (*Linum usitatisimum* L.)—A plant system for study of embryogenesis. Acta Biol. Cracoviensia Ser. Bot..

[25] Zeng F.C., Zhang X.L., Jin S.X., Cheng L., Liang S.G., Hu L.S., Guo X.P., Nie Y.C., Cao J.L. (2007). Chromatin reorganization and endogenous auxin/cytokinin dynamic activity during somatic embryogenesis of cultured cotton cell. Plant Cell Tissue Organ Cult..

[26] Testillano P.S., Gómez-Garay A., Pintos B., Risueño M.C., Loyola-Vargas V., Ochoa-Alejo N. (2018). Somatic Embryogenesis of *Quercus suber* L. from Immature Zygotic Embryos. Plant Cell Culture Protocols. Methods in Molecular Biology.

[27] Mauri P., Manzanera J. (2003). Induction, maturation and germination of holm oak (*Quercus ilex* L.) somatic embryos. Plant Cell Tissue Organ Cult..

[28] Lebtahi F., Errahmani M., Nadia B. (2015). Propagation of Cork oak (*Quercus suber* L.) by axillary shoot and somatic embryogenesis. Propag. Ornam. Plants.

[29] Hernández I., Celestino C., Toribio M. (2003). Vegetative propagation of *Quercus suber* L. by somatic embryogenesis. Plant Cell Rep..

[30] Pinto G., Valentim H., Costa A., Castro S., Santos C. (2002). Somatic embryogenesis in leaf callus from a mature *Quercus suber* L. Tree. Vitr. Cell. Dev. Biol.-Plant.

[31] Sané D., Aberlenc-Bertossi F., Diatta L., Guèye B., Daher A., Sagna M., Duval Y., Borgel A. (2012). Influence of Growth Regulators on Callogenesis and Somatic Embryo Development in Date Palm (*Phoenix dactylifera* L.) Sahelian Cultivars. Sci. World J..

[32] Taylor N., Edwards M., Kiernan R., Davey C., Blakesley D., Henshaw G. (1996). Development of friable embryogenic callus and embryogenic suspension culture systems in cassava (*Manihot esculenta* Crantz). Nat. Biotechnol..

[33] Lee K., Jeon H., Kim M. (2002). Optimization of a mature embryo-based in vitro culture system for high-frequency somatic embryogenic callus induction and plant regeneration from *japonica* rice cultivars. Plant Cell Tissue Organ Cult..

[34] Carman J.G., Jefferson N.E., Campbell W.F. (1987). Induction of embryogenic *Triticum aestivum* L. calli. I. Quantification of genotype and culture medium effects. Plant Cell Tissue Organ Cult..

[35] Gomes da Cunha A.C., Fernandes Ferreira M. (1996). Somatic embryogenesis, organogenesis and callus growth kinetics of flax. Plant Cell Tissue Organ Cult..

[36] Guo G., Jeong B.R. (2021). Explant, Medium, and Plant Growth Regulator (PGR) Affect Induction and Proliferation of Callus in *Abies koreana*. Forests.

[37] Cheng W.H., Wang F.L., Cheng X.Q., Zhu Q.H., Sun Y.Q., Zhu H.G., Sun J. (2015). Polyamine and Its Metabolite H_2_O_2_ Play a Key Role in the Conversion of Embryogenic Callus into Somatic Embryos in Upland Cotton (*Gossypium hirsutum* L.). Front. Plant Sci..

[38] Haddadi P., Moieni A., Karimzadeh G., Abdollahi M.R. (2008). Effects of Gibberellin, Abscisic Acid and Embryo Desiccation on Normal Plantlet Regeneration, Secondary Embryogenesis and Callogenesis in Microspore Culture of *Brassica napus* L. cv. PF_704_. Int. J. Plant Prod..

[39] Loureiro J., Pinto G., Lopes T., Doležel J., Santos C. (2005). Assessment of ploidy stability of the somatic embryogenesis process in *Quercus suber* L. using flow cytometry. Planta.

[40] Maâtaoui M., Espagnac H., Michaux-Ferrière N. (1990). Histology of Callogenesis and Somatic Embryogenesis Induced in Stem Fragments of Cork Oak (*Quercus suber*) Cultured In Vitro. Ann. Bot..

[41] Conner A.J., Searle H., Jacobs J.M.E. (2019). Rejuvenation of chicory and lettuce plants following phase change in tissue culture. BMC Biotechnol..

[42] Karami O., Aghavaisi B., Mahmoudi-Pour A. (2009). Molecular aspects of somatic-to-embryogenic transition in plants. J. Biol. Chem..

[43] Gulzar B., Mujib A., Malik M.Q., Sayeed R., Mamgain J., Ejaz B. (2022). Genes, proteins and other networks regulating somatic embryogenesis in plants. J. Genet. Eng. Biotechnol..

[44] Meireles B., Usié A., Barbosa P., Fortes A.M., Folgado A., Chaves I., Carrasquinho I., Costa R.L., Gonçalves S., Teixeira R.T. (2018). Characterization of the cork formation and production transcriptome in *Quercus cerris*  × * suber* hybrids. Physiol. Mol. Biol. Plants.

[45] Sobral R., Costa M.M.R. (2017). Role of floral organ identity genes in the development of unisexual flowers of *Quercus suber* L. *Sci*. Rep..

[46] Capote T., Usié A., Barbosa P., Romos M., Morais-Cecílio L., Gonçalves S. (2019). Transcriptome dynamics of cork oak (*Quercus suber*) somatic embryogenesis reveals active gene players in transcription regulation and phytohormone homeostasis of embryo development. Tree Genet. Genomes.

[47] Zhang B.X., Zhang H.L., Xia Y.J. (2024). Harnessing spatial transcriptomics for advancing plant regeneration research. Trends Plant Sci..

[48] Czernicka M., Chłosta I., Kęska K., Kozieradzka-Kiszkurno M., Abdullah M., Popielarska-Konieczna M. (2021). Protuberances are organized distinct regions of long-term callus: Histological and transcriptomic analyses in kiwifruit. Plant Cell Rep..

[49] Chalupa V., Jain S.M., Gupta P.K., Newton R.J. (1995). Somatic Embryogenesis in Oak (*Quercus* spp.). Somatic Embryogenesis in Woody Plants.

[50] Salaün C., Lepiniec L., Dubreucq B. (2021). Genetic and Molecular Control of Somatic Embryogenesis. Plants.

[51] Li Y., Yan L.J., Wang H.H., Tang S.J. (2025). Plant regeneration through somatic embryogenesis in *Tilia cordata*. Plant Cell Tissue Organ Cult..

[52] Bhat A.Y., Shahzad A., Kausar A., Rashid A. (2024). Integrating leaf and root induced shoot regeneration and embryogenesis for the conservation of *Atropa acuminata* Royle ex Lindl—An endangered Himalayan herb. Plant Cell Tissue Organ Cult..

[53] Liang H.Z., Xiong Y.P., Guo B.Y., Yan H.F., Jian S.G., Ren H., Zhang X.H., Li Y., Zeng S.J., Wu K.L. (2020). Shoot organogenesis and somatic embryogenesis from leaf and root explants of *Scaevola sericea*. Sci. Rep..

[54] Akkenapally S., Mudalkar S., Bodiga S., Sundaram R., Bokam A.K. (2024). In vitro callus generation and somatic embryogenesis from leaf explant of *Madhuca longifolia var latifolia* (Roxb.) A. Chev. Vegetos.

[55] Ku S.S., Woo H.A., Shin M.J., Jie E.Y., Kim H., Kim H.S., Cho H.S., Jeong W.J., Lee M.S., Min S.R. (2023). Efficient Plant Regeneration System from Leaf Explant Cultures of *Daphne genkwa* via Somatic Embryogenesis. Plants.

[56] Zhai M., Dai Q., Zhao Y., Zhang S.Y., Yang Y.L. (2025). Induction of callus and establishment of suspension culture system in *Cassia mimosoides herb*. Vitr. Cell. Dev. Biol.-Plant.

[57] Duong T.N., Nguyen T.M., Pham Q.T., Le T.M., Nguyen T.H., Ngo C.C., Nguyen H.N., Vinh D.N. (2006). Liquid culture as a positive condition to induce and enhance quality and quantity of somatic embryogenesis of *Lilium longiflorum*. Sci. Hortic..

[58] Ibraheem Y., Pinker I., Böhme M. (2013). A comparative study between solid and liquid cultures relative to callus growth and somatic embryo formation in *Phoenix dactylifera* L. cv. Zaghlool. Emir. J. Food Agric..

[59] Naouar B.A., Rajae B., Safaa R., Ouafaa H., Ibtissam B., Mustapha H., Latifa A., Alain B., Patrick M., Ahmed L. (2023). Influence of exogenous polyamines on the secondary somatic embryogenesis of cork oak (*Quercus suber* L.). Bioengineered.

[60] Fernández-Guijarro B., Celestino C., Toribio M. (1995). Influence of external factors on secondary embryogenesis and germination in somatic embryos from leaves of *Quercus suber*. Plant Cell Tissue Organ Cult..

[61] Fan Y.P., Tang Z.M., Wei J.M., Yu X.M., Guo H.H., Li T.T., Guo H.X., Zhang L., Fan Y.J., Zhang C.Y. (2022). Dynamic Transcriptome Analysis Reveals Complex Regulatory Pathway Underlying Induction and Dose Effect by Different Exogenous Auxin IAA and 2,4-D During in vitro Embryogenic Redifferentiation in Cotton. Front. Plant Sci..

[62] Zhang X.L., Wang Y.L., Yan Y.Y., Peng H., Long Y., Zhang Y.C., Jiang Z., Liu P., Zou C.Y., Peng H.W. (2019). Transcriptome sequencing analysis of maize embryonic callus during early redifferentiation. BMC Genomics.

[63] Wójcikowska B., Chwiałkowska K., Nowak K., Citerne S., Morończyk J., Wójcik A.M., Kiwior-Wesołowska A., Francikowski J., Kwaśniewski M., Gaj M.D. (2024). Transcriptomic profiling reveals histone acetylation-regulated genes involved in somatic embryogenesis in *Arabidopsis thaliana*. BMC Genomics.

[64] Avilez-Montalvo J.R., Quintana-Escobar A.O., Méndez-Hernández H.A., Aguilar-Hernández V., Brito-Argáez L., Galaz-Ávalos R.M., Uc-Chuc M.A., Loyola-Vargas V.M. (2022). Auxin-Cytokinin Cross Talk in Somatic Embryogenesis of *Coffea canephora*. Plants.

[65] Méndez-Hernández H.A., Quintana-Escobar A.O., Uc-Chuc M.A., Loyola-Vargas V.M. (2021). Genome-Wide Analysis, Modeling, and Identification of Amino Acid Binding Motifs Suggest the Involvement of GH3 Genes during Somatic Embryogenesis of *Coffea canephora*. Plants.

[66] Yu X.N., Lu M.J., Zhou M., Wang H.Y., Feng J.Y., Wen Y.Q. (2023). Reduction of pectin may decrease the embryogenicity of grapevine (*Vitis vinifera*) pro-embryonic masses after 10 years of in vitro culture. Sci. Hortic..

[67] Pérez-Pérez Y., Carneros E., Berenguer E., Solís M.-T., Bárány I., Pintos B., Gómez-Garay A., Risueño M.C., Testillano P.S. (2019). Pectin De-methylesterification and AGP Increase Promote Cell Wall Remodeling and Are Required During Somatic Embryogenesis of *Quercus suber*. Plant Sci..

[68] Subrahmanyeswari T., Gantait S., Sarkar R., Kamble S.N., Singh S., Bhattacharyya S. (2024). Polyamines- and growth inducers-mediated enhanced mono-phasic in vitro regeneration of sugar leaf plant (*Stevia rebaudiana* Bert.) in liquid medium. S. Afr. J. Botan..

[69] Sanaa N., Frédéric L., Françoise G., Dominique L., Marc F., Alain D., Annie J. (1999). Polyamine Content and Somatic Embryogenesis in *Papaver somniferum* Cells Transformed with sam-1 Gene. J. Plant Physiol..

[70] Zhang C., Xu X., Xu X., Li Y., Zhao P., Chen X., Shen X., Zhang Z., Chen Y., Liu S. (2022). Genome-wide identification, evolution analysis of cytochrome P450 monooxygenase multigene family and their expression patterns during the early somatic embryogenesis in *Dimocarpus longan* Lour. Gene.

[71] Lan J.Q., Wang N., Wang Y.T., Jiang Y.D., Yu H., Cao X.F., Qin G.J. (2023). Arabidopsis TCP4 transcription factor inhibits high temperature-induced homeotic conversion of ovules. Nat. Commun..

[72] Li Z.Y., Zhang D.P., Shi P., Htwe Y.M., Yu Q., Huang L.Y., Zhou H.Q., Liu L.Y., Wang Y. (2023). Cell wall lignification may be necessary for somatic embryogenesis of areca palm (*Areca catechu*). Sci. Hortic..

[73] Xie Q., Ahmed U., Qi C., Du K., Luo J., Wang P., Zheng B., Shi X. (2024). A protocol for identifying universal reference genes within a genus based on RNA-Seq data: A case study of poplar stem gene expression. For. Res..

[74] Shi X.P., Zhang C.J., Liu Q.H., Zhang Z., Zheng B., Bao M.Z. (2016). De novo comparative transcriptome analysis provides new insights into sucrose induced somatic embryogenesis in camphor tree (*Cinnamomum camphora* L.). BMC Genomics.

[75] Teng M., Love M.I., Davis C.A., Djebali S., Dobin A., Graveley B.R., Li S., Mason C.E., Olson S., Pervouchine D. (2016). A benchmark for RNA-seq quantification pipelines. Genome Biol..

[76] Wang Z., Lyu Z., Pan L., Zeng G., Randhawa P. (2019). Defining housekeeping genes suitable for RNA-seq analysis of the human allograft kidney biopsy tissue. BMC Med. Genomics.

[77] Chen Y., Li X., Su L., Chen X., Zhang S.T., Xu X.P., Zhang Z.H., Chen Y.K., Xu X.H., Lin Y.L. (2018). Genome-wide identification and characterization of long non-coding RNAs involved in the early somatic embryogenesis in *Dimocarpus longan* Lour. BMC Genomics.

[78] Gao Y., Cui Y., Zhao R., Chen X., Zhang J., Zhao J., Kong L. (2022). Cryo-Treatment Enhances the Embryogenicity of Mature Somatic Embryos via the lncRNA–miRNA–mRNA Network in White Spruce. Int. J. Mol. Sci..

[79] Ok R.L., Ngoc Q.N., Kwang H.L., Young C.K., Jiho S. (2017). Cytohistological study of the leaf structures of *Panax ginseng* Meyer and *Panax quinquefolius* L.. J. Ginseng Res..

[80] Li X., Huang X., Wen M., Wen Y., Chen Y.M., Liu Y.L., Liu X.D. (2023). Cytological observation and RNA-seq analysis reveal novel miRNAs high expression associated with the pollen fertility of neo-tetraploid rice. BMC Plant Biol..

[81] Andrews S. (2010). FastQC: A Quality Control Tool for High Throughput Sequence Data. http://www.bioinformatics.babraham.ac.uk/projects/fastqc/.

[82] Love M.I., Huber W., Anders S. (2014). Moderated estimation of fold change and dispersion for RNA-seq data with DESeq2. Genome Biol..

[83] Ebadzad G., Cravador A. (2014). Quantitative RT-PCR analysis of differentially expressed genes in *Quercus suber* in response to *Phytophthora cinnamomi* infection. SpringerPlus.

[84] Lalitha S. (2000). Primer premier 5. Biotech Softw. Internet Rep. Comput. Softw. J. Sci..

[85] Livak K.J., Schmittgen T.D. (2001). Analysis of relative gene expression data using real-time quantitative PCR and the 2^−ΔΔCT^ method. Methods.

[86] Winston W. (2011). Microsoft Excel 2010 Data Analysis and Business Modeling.

[87] Sweet S.A., Karen G.M. (1999). Data Analysis with SPSS..

[88] Swift M.L. (1997). GraphPad prism, data analysis, and scientific graphing. J. Chem. Inf. Comput. Sci..

[89] Edwards P.M. (2002). Origin 7.0: Scientific graphing and data analysis software. J. Chem. Inf. Comput. Sci..

[90] R Core Team (2018). R: A Language and Environment for Statistical Computing.

